# Supplementary material for: Dietary Natural Compounds and Vitamins as Potential Cofactors in Uterine Fibroids Growth and Development
Source: Nutrients. 2022 Feb 9;14(4):734. doi: 10.3390/nu14040734 (PMC8880543; doi:10.3390/nu14040734)
Supplement: Supplementary file 1 [file nutrients-14-00734-s001.zip › nutrients-1576169-supplementary.pdf]

| Authors<br>Year and country of publication; Evidence level | Dietary compounds<br>Kind of intervention | Kind of population/animal model/ in-vitro study | Effects on fibroids<br>(Conclusions) | Comments |
|---|---|---|---|---|
| **Carotenoids; Vitamins** | | | | |
| Sahin et al<br>2004<br>Japan; L 5 | **Lycopene-** supplementation with 100 mg or 200 mg Lycopene per kilogram of diet | Animal model- Japanese quail<br>No 120 | *Dietary supplementation with lycopene ↓the incidence of spontaneously occurring leiomyomas of the oviduct | *Higher doses of lycopene than in normal human diet |
| Broaddus et al.<br>2004<br>United States; L 5 | doses for 4-HPR (synthetic **retinoid analogue**) and DFMO (alpha-difluoromethylornithine) were 1 µmol/L and 1 mmol/L, respectively | in-vitro study - primary cultures of human uterine leiomyomas | * 4-HPR and DFMO cause ↓ leiomyoma growth (by the modulation of ECM, the induction of p53, p21, and p16)<br>* DFMO and 4-HPR ↑ an apoptosis | |
| Sahin et al<br>2007<br>Japan; L 5 | **Lycopene-** diet supplemented with 25 g or 50 g of tomato powder per kg of diet (0.8 mg lycopene per g of tomato powder). | Animal model- Japanese quail<br>No 150 | * Dietary supplementation with tomato powder ↓ the incidence and size of leiomyoma of the oviduct | |
| Terry et al.<br>2008 (10 years follow up)<br>United States Nurses' Health Study II; L 2 | food frequency questionnaire (FFQ) regarding **lycopene** and **beta-carotene** intake | 8212 premenopausal women (ages 26–46) | * Lycopene intake not associated with the UFs risk<br>* High intake of beta-carotene slightly ↑ the risk of UFs (only in current smokers) | *Fibroids confirmed by an USG or hysterectomy (based on questionnaires) |
| Bläuer et al.<br>2009<br>Finland; L 5 | different concentrations of **$1,25(OH)_2D_3$** (0.1 nM; 1 nM, 10 nM, 100 nM, 500 nM, 1000 nM) | in-vitro study-the human leiomyoma cell culture | * $1,25(OH)_2D_3$ ↓ the growth of leiomyoma cells in a concentration-dependent manner (after the 1000 nM ↓approximately 50%)<br>* Suppression of leiomyoma cells already detected in the physiologic vitamin D levels<br>* Hypovitaminosis D plays an important role in the etiology and growth of leiomyoma | *VDR was present in all leiomyoma specimens (immunohistochemical analysis) |
| Sharan et al.<br>2011<br>United States; L 5 | **$1,25(OH)_2D_3$** treatment with different doses (1 nM, 10 nM, 100 nM, and 1,000 nM) | in-vitro study-the human leiomyoma cell line | * $1,25(OH)_2D_3$ ↓ growth of HuLM cells (↓ the expression of PCNA and CDK1; ↓ anti-apoptotic BCL-2 and BCL-w)<br>* $1,25(OH)_2D_3$ ↓ *COMT* mRNA and protein expression and enzyme activity in HuLM cells | |
| Martin et al.<br>2011 (2003-2004)<br>United States National Health and Nutrition Examination Survey (NHANES); L 2 | B- carotene, folate,<br>vitamin A (retinols), vitamin B6, vitamin B12, vitamin C, vitamin E. | 887 premenopausal women (aged 20–49) | * Statistically significant dose-response positive relationship between vitamin A and uterine fibroids risk<br>* Dose-responsive positive relationship between vitamin C, vitamin E and uterine fibroids (statistically insignificant)<br>* Statistically insignificant, positive relationship between b-carotene and uterine fibroids | *Micronutrient levels measured in serum samples women with uterine fibroids<br>*Fibroids self-reported during the in-home interview (on the base of previous doctor's diagnosis) |

| | | | | |
|---|---|---|---|---|
| Halder et al.<br>2011<br>United States; L 5 | different concentrations of **1,25(OH)$_2$D$_3$** (0.1 or 1 μM) | in-vitro study-the human leiomyoma cell line | * 1,25(OH)$_2$D$_3$ is an antifibrotic factor in human leiomyoma cells (↓ TGF-β3 induced fibronectin, plasminogen activator inhibitor-1 and collagen type 1 protein expression)<br>* 1,25(OH)$_2$D$_3$ ↓TGF-β3 and TGF-β3-mediated effects | |
| Halder et al.<br>2012<br>United States; L 5 | **1,25(OH)$_2$D$_3$** (0.5 μg/kg per day) subcutaneously for 3 weeks | Animal model- female Eker rats randomized into two groups:<br>1) the control group (No 6)<br>2) the treatment group (No 6) – treated with 1,25(OH)$_2$D$_3$ | * Treatment with 1,25(OH)$_2$D$_3$ significantly ↓ leiomyoma tumor size<br>* Treatment with 1,25(OH)$_2$D$_3$ ↓cell growth and proliferation-related genes (PCNA, cyclin D1, Myc*,* Cdk1*,* Cdk2, *and* Cdk4), antiapoptotic genes (*Bcl2* and *Bcl2l1*), and estrogen and progesterone receptors<br>* 1,25(OH)$_2$D$_3$ ↓ expression of PCNA and MKI67 (markers of proliferation) and ↑ expression of caspase 3 | |
| Halder et al.<br>2013<br>United States; L 5 | **1,25(OH)$_2$D$_3$** treatment in various concentrations (1-1000 nM). | in vitro- uterine fibroid cell cultures (surgically removed fibroids used to generate primary uterine fibroid cells) | * 1,25(OH)$_2$D$_3$ significantly ↓ mRNA levels of MMP-2 and MMP-9 in a concentration-dependent manner<br>* 1,25(OH)$_2$D$_3$ ↓mRNA levels of MMP-1, MMP-3, MMP-13, MMP-14 in HuLM cells<br>* 1,25(OH)$_2$D$_3$ significantly ↓ MMP-2 and MMP-9 protein levels in a concentration-dependent manner in HuLM<br>* 1,25(OH)$_2$D$_3$ ↑ the mRNA levels of vitamin D receptor (VDR) and TIMP-2 in a concentration-dependent manner in HuLM cells | |
| Halder et al.<br>2013<br>United States; L 5 | treatment with increasing concentrations of **1,25(OH)$_2$D$_3$** (0, 1, 10, 100, 1000 nM) for 48 h | in-vitro study- cultured immortalized human uterine fibroid cell line (HuLM) | *1,25(OH)$_2$D$_3$ can sensitize HuLM cells and induce VDR protein expression in HuLM cells<br>* 1,25(OH)$_2$D$_3$ ↓the presence of disorganized collagen type 1, excessive fibronectin protein expression, PAI-1 protein expression and proteoglycans expression in HuLM cells<br>* 1,25(OH)$_2$D$_3$ in HuLM cells ↓ the synthesis and accumulation of disorganized actin fibers | |
| Halder et al.<br>2014<br>United States; L 5 | **paricalcitol** and **1,25(OH)$_2$D$_3$** | in-vitro study: Eker rat-derived uterine leiomyoma cell line<br>Animal model- nude mice randomized into 3 groups: | *Paricalcitol ↓ proliferation of the immortalized human uterine fibroid cells<br>* Paricalcitol and 1,25(OH)$_2$D$_3$ significantly ↓fibroid tumor size | |

| | | | | |
|---|---|---|---|---|
| | | Control; Paricalcitol (300 ng/kg/d for 4 weeks); Vit D3 group (500 ng/kg/d for 4 weeks) | * Fibroid shrinkage slightly higher in the paricalcitol-treated group | |
| Al-Hendy et al.<br>2015<br>United States; L 5 | Treatment with increasing doses of **$1,25(OH)_2D_3$** (0, 10, 100, and 1000 nᴍ) | in-vitro study - Human uterine leiomyoma (HuLM) cells | * $1,25(OH)_2D_3$ significantly ↓ the estrogen-induced proliferation of HuLM cells<br>* Treatment of $1,25(OH)_2D_3$ ↓the expression of nuclear ER-α, PR-A, PR-B, and the nuclear steroid receptor coactivator (SRC) family members in HuLM cells<br>* $1,25(OH)_2D_3$ induced its own VDR in a dose- and time-dependent manner in HuLM cells | *Higher levels of ER-α, PR-A, or PR-B and reduced levels of VDR found in human UFs |
| Al-Hendy et al.<br>2016<br>United States; L 5 | Treatment with increasing concentrations of **$1,25(OH)_2D_3$** | in-vitro study - Immortalized human UF cells (HuLM) and human primary UF (PUF) cells | * $1,25(OH)_2D_3$ administration ↓the levels of Wnt4 and β-catenin in both HuLM and PUF cells<br>* $1,25(OH)_2D_3$ ↓ the expression/activation of mTOR signaling in both cell types | *UF tumors with Med 12 mutations showed an up-regulation of Wnt4 and β-catenin (play a major role in fibroid pathogenesis) |
| Ciavattini et al.<br>2016<br>Italy; L 2 | supplementation therapy with 50,000 IU of **$1,25(OH)_2D_3$** (oral solution) once per week for 8 weeks, followed by maintenance therapy of 2000 IU daily for a year. | 108 women with uterine fibroids and hypovitaminosis D:<br>study group: 53 patients with Vit D therapy<br>control group: 55 women | * Negative correlation between the baseline $25(OH)D_3$ concentration and the volume of the largest fibroid and the total volume of fibroids<br>* No correlation between the baseline $25(OH)D_3$ levels and the number of fibroids.<br>* $1,25(OH)_2D_3$ supplementation ↓ the progression of fibroids and related symptoms and ↓ the need of surgical or medical therapy | *Hypovitaminosis D defined as a $25(OH)D_3$ serum level $< 30$ ng/mL |
| Elhusseini et al.<br>2018<br>United States; L 5 | vitamin D-deficient diet | Animal model: mouse model<br>Female mice divided into two groups:<br>control (n = 10) fed on normal diet containing vit D; vitamin D-deficient diet group (n = 10)- totally lacking vit D for 8 weeks. | Vitamin D deficiency:<br>* ↑ expression of sex steroid receptors in murine myometrium<br>* ↑ expression of proliferation related genes<br>* promotes the fibrosis<br>* ↑ inflammation in murine myometrium<br>* ↑ DNA damage in murine myometrium | |
| Corachán et al.<br>2019<br>Spain; L 5 | **$1,25(OH)_2D_3$** in various concentrations (10 nM, 100 nM, and 1,000 nM) | Human uterine leiomyomas (No 22) | * $1,25(OH)_2D_3$ therapy effective in stabilizing leiomyoma size and preventing its growth (induced cell growth arrest and ↓proliferation in HuLM cells; VitD ↓ Wnt-pathway expression in UL cells at gene and protein levels)<br>* $1,25(OH)_2D_3$ did not induce apoptosis expression | *Uterine leiomyomas collected from premenopausal women aged 35−52 years undergoing surgery |

| | | | | |
|---|---|---|---|---|
| Ali et al.<br>2019<br>United States; L 5 | treatment with **1,25(OH)$_2$D$_3$**<br>100 nM for 3 d or not treated | cultured HuLM cells (human fibroid obtained from women of reproductive age (aged 22–55 years) undergoing hysterectomy or myomectomy) | *Vitamin D receptor (VDR) expression is decreased in HuLM cells and induces DNA damage accumulation and DNA damage response defects in HuLM cells<br>* 1,25(OH)$_2$D$_3$ treatment suppresses DNA damage and restores the DNA damage response via induction of VDR in HuLM cells<br>* 1,25(OH)$_2$D$_3$ treatment ↓UF formation through the amelioration of pathogenic DNA damage | *Vitamin D3/VDR axis is functionally linked to DNA damage and instability status in UF cells |
| Hajhashemi et al.<br>2019<br>Iran; L 1 | oral administration of **1,25(OH)$_2$D$_3$** (50,000 IU every 2 weeks for 10 weeks) or placebo (randomly selected) | 69 participants with uterine leiomyomas and vitamin D deficiency (age 35 - 49 years):<br>Study group (n=35) - vitamin D<br>Placebo group (n=34)- placebo | * Leiomyomas size in vitamin D$_3$ group was significantly ↓ as compared to placebo<br>* No toxicity observed after 1,25(OH)$_2$D$_3$ | *In USG: 1-2 uterine leiomyomas, size 20-80 mm<br>*Serum levels of 25(OH)D$_3$ <20 ng/ml |
| Xess et al.<br>2020<br>India; L 2 | therapy with 60,000 IU of **1,25(OH)$_2$D$_3$** (oral solution) once per week for 12 weeks, followed by maintenance therapy of 2000 IU weekly for a year. | 110 premenopausal women (mean age 45 years) with fibroids<br>Study group (n=60)<br>Control group (n=50) | * After 1,25(OH)$_2$D$_3$ treatment no increase or decrease in size or number of UF tumors<br>* Without supplementation slight but significant ↑ in size of UFs and need for subsequent medical or surgical therapy | *Fibroids diagnosed with TV- US (>1 uterine fibroid; mean diameter 1-5 cm) |
| Arjeh et al.<br>2020<br>Iran; L 1 | 50,000 IU oral **1,25(OH)$_2$D$_3$** tablets or placebo (lactose sugar) tablets for 12 weeks (patients randomly assigned) | 60 women (aged 22–40 years) with UFs and vitamin D deficiency:<br>Study group (n = 30)- vitamin D<br>Control group (n = 30)- placebo | * 1,25(OH)$_2$D$_3$ therapy ↓ growth of uterine fibroids<br>* Statistically insignificant ↓ in the volume of fibroids observed after 1,25(OH)$_2$D$_3$ therapy<br>* Significant ↑in the fibroids' size observed in the placebo group after 12 weeks | *Vitamin D deficiency: 25(OH)D$_3$ <30 ng/mL<br>*UFs confirmed in abdominal or transvaginal USG |
| Corachán et al.<br>2020<br>Spain; L 5 | two doses (0.5 µg/kg/d and 1 µg/kg/d) of short-term (21 days) and long-term (60 days) **1,25(OH)$_2$D$_3$** treatment. | Animal model- human leiomyomas collected from patients were implanted in ovariectomized NOD-SCID mice. | * High-dose and long-term treatment with 1,25(OH)$_2$D$_3$ induced a significant ↓ in leiomyoma size<br>* Cell proliferation significantly ↓ after long term and high dose treatment with 1,25(OH)$_2$D$_3$ (not decreased after short term treatment)<br>* Collagen-I and plasminogen activator inhibitor 1 significantly ↓ by long-term high-dose 1,25(OH)$_2$D$_3$ treatment<br>* Long-term, high-dose 1,25(OH)$_2$D$_3$ significantly ↓ TGF-β3 expression<br>* Apoptosis significantly ↑ with short- and long-term high-dose 1,25(OH)$_2$D$_3$ treatment | |

| | | | | |
|---|---|---|---|---|
| Corachán et al.<br>2021<br>Spain; L 5 | **$1,25(OH)_2D_3$** treatment | human leiomyomas (collected from patients) | * $1,25(OH)_2D_3$ treatment ↓ Wnt/β-catenin and TGFβ pathways in HULP cells in MED12-mutated leiomyomas and wild-type leiomyomas<br>* $1,25(OH)_2D_3$ treatment ↓ proliferation and ECM formation in different molecular subtypes of uterine leiomyomas | |
| Davari Tanha et al.<br>2021<br>Iran; L 1 | treatment with **$1,25(OH)_2D_3$**<br>50,000 IU for two months. | Study group- 110 patients treated with vit D<br>Control group (No 110) - no treatment | * $1,25(OH)_2D_3$ in women with hypovitaminosis D ↓ the leiomyoma growth | *≥1 uterine fibroid (myoma size measured by ultrasound) and<br>*$25(OH)D_3$ level: 20-30 ng/ml. |
| Suneja et al.<br>2021<br>India; L 1 | **$1,25(OH)_2D_3$** in dose of 60,000 IU weekly for 8 weeks followed by 60,000 IU every 2 weeks for 8 weeks. | 30 premenopausal women with uterine leiomyoma and concomitant hypovitaminosis D (<30 ng/ml) | * Significant negative correlation between the baseline $25(OH)D_3$ and leiomyoma volume<br>* Significant ↓ of leiomyoma-related symptoms observed at 16 weeks of therapy<br>* $1,25(OH)_2D_3$ therapy effective in stabilizing uterine and leiomyoma volume (insignificant ↓ the mean uterine and leiomyoma volume) | |
| Elkafas et al.<br>2021<br>Egypt/United States; L 5 | **$1,25(OH)_2D_3$** treatment in various concentrations (100 and 500 nM) | Animal model- the Eker rat model | * $1,25(OH)_2D_3$ treatment significantly ↓the DNA damage levels in myometrial stem cells (MMSCs)<br>* the levels of MRN complex (key DNA damage repair members) ↑ in MMSCs after $1,25(OH)_2D_3$<br>* $1,25(OH)_2D_3$ acts on DNA repair via the MRN complex/ATM axis, restores the DNA repair signaling network and enhances DNA damage response (DDR) | *$1,25(OH)_2D_3$ treatment useful in reversing the action of EDCs |
| Wise et al.<br>2021 (5-year study)<br>United States; L 1 | **Lycopene** and other carotenoids, Vitamin A only from diet | African American women<br>Age 23-35; No 1230 | *Lycopene**,** carotenoids and vitamin A from diet had no effect on UFs | *Only presence -not estimated size, growth and location |
| **Dairy products, Fruits and vegetables** | | | | |
| Wise et al.<br>2010<br>United States; L 2 | food frequency questionnaire (FFQ) regarding **dairy c**onsumption (milk, cream, ice cream yogurt, cheese and butter). | 5 871 participants (aged 21–69 years) from premenopausal US Black Women's Health Study | *High dairy intake inversely associated with uterine leiomyomata risk among black women | *UFs diagnosed by ultrasound or surgery |
| Wise et al.<br>2011 (1997-2009)<br>United States; L 2 | **Fruit; vegetables** (Yellow-orange vegetables: carrots, tomatoes or tomato juice, sweet potatoes; cruciferous vegetables: broccoli, collard and mustard greens, cabbage; green leafy vegetables: | 22 583 African American premenopausal women (aged 21–69 years) | * Fruit and vegetable intake inversely associated with UFs (association stronger for fruit than for vegetables)<br>* Inverse association between dietary vitamin A intake and the risk of UFs | *UFs diagnosed in 6627 cases:<br>by ultrasonography (*n* = 4346) or surgery (*n* = 2281) |

| | | | | |
|---|---|---|---|---|
| | spinach, green salad), carotenoids, folate, fiber, and vitamins A, C, and E | | (predominantly preformed vitamin A from animal sources (liver, breakfast cereals and milk), not provitamin A from fruit and vegetable sources)<br>* UF risk not associated with dietary intake of vitamins C and E, folate, fiber and carotenoids, including lycopene | |
| He et al.<br>2013 (2-year study)<br>China; L 3 | **fruit and vegetable** intake:<br>low (never/<1 day/week),<br>intermediate (1-2 days/week),<br>high (≥3 days/week) | premenopausal and postmenopausal women<br>No 283 (n=73 study group and n=210 controls) | ***** Vegetable and fruit intake significantly ↓ the risk of UFs in premenopausal women<br>* Insignificant UFs risk reduction in postmenopausal women after vegetable and fruit intake | *UFs confirmed by USG or hysterectomy<br>*Only presence (not estimated size, growth and location) |
| Wise et al.<br>2013<br>United States; L 2 | **Dairy** items (milk, cream, ice cream yogurt, cheese and butter) consumption frequencies ranging from “never or <1 per month” to “≥6 per day” (self-administered questionnaires) | Black Women's Health Study<br>59 000 African-American women (aged 21–69 year) | * Inverse association between high dairy intake and UFs among African Americans | *Questionnaire- uterine fibroids, confirmed by ultrasound or surgery<br>*Dairy intake is lower among black than white Americans |
| Shen et al.<br>2016 (2-year study)<br>China; L 3 | **fruit and vegetables** | 1200 Chinese women age 23-35 (600 women with uterine fibroids in study group and<br>600 patients without fibroids in control group). | * Fresh fruits and cruciferous vegetables may reduce the incidence of UFs<br>* Drinking green tea was unrelated to risk of UFs<br>* Dairy consumption have preventative effects | *Study group- Uterine myomas confirmed by histopathologic examination<br>*Control group - Uterine myomas excluded by ultrasounds |
| Zhou et al.<br>2020 (1-year study)<br>China; L 2 | **grain cereals, legumes, vegetables, fruits** (i.e. apple orange, peach, mandarin,grape, banana, kiwi fruit, melon watermelon), nuts, dairy products (liquid milk and yoghurt), meats, fish and eggs | urban premenopausal women (age 20-45) No 248 | ***** UFs risk ↓ significantly with increased intake of vegetables and nuts<br>* Meat/fish and fruits: no correlation with UFs<br>* Carrot, kiwi fruit, and seaweed: inversely associated with the risk of UFs<br>* Yoghurt: inverse association with the risk of UFs | *Uterine myomas confirmed by ultrasounds, pelvic exam or during surgery |
| Orta et al.<br>2020 (18- year study) United States; L 2 | The frequency of **dairy** consumption:<br>(≤4/week- >4/day)<br><br>Yogurt consumption:<br>(≤4/week- 2+/day) | No 81 590 premenopausal women (aged 25-42 at baseline) | * Dairy consumption not associated with UFs risk (except calcium from foods and yoghurt)<br>* High consumption of yoghurt and calcium from foods: inverse association with UFs risk | *Uterine myomas confirmed by ultrasounds or hysterectomy |
| **Plant-derived compounds and microelements** | | | | |

| | | | | |
|---|---|---|---|---|
| Ozercan et al.<br>2008<br>Turkey; L 5 | supplementation with 200 or 400 mg of epigallocatechin gallate **(EGCG)**/kg of diet | Animal model- Japanese quail<br>No 180 | * EGCG supplementation ↓ the incidence, number and size of spontaneously occurring leiomyoma<br>* Serum malondialdehyde and TNF-α concentrations ↓ after EGCG supplementation | |
| Malik et al.<br>2009<br>United States; L 5 | **Curcumin** in various concentrations | Immortalized leiomyoma cells exposed to concentrations of curcumin ranging from 5 to 40 µM | * Curcumin showed an antiproliferative effect on leiomyoma cell lines<br>* Curcumin regulated leiomyocyte apoptosis via stimulation caspase-3 and caspase-9 expression and inhibition extracellular signal-regulated kinase 1 (ERK 1), ERK 2, and nuclear factor kappa B (NF-κB)<br>* Curcumin inhibited expression of fibronectin (ECM component) in leiomyoma cells | |
| Zhang et al.<br>2010<br>United States; L 5 | green tea extract-epigallocatechin gallate **(EGCG)** | in-vitro study- leiomyoma cells culture treated with EGCG in various concentrations (0, 0.1, 1.0, 10, 50, 100 and 200µM) | * EGCG ↓ the proliferation (in a dose- and time-dependent manner)<br>* EGCG induced apoptosis in cultured human leiomyoma cells (at ≥50µM significantly ↓ the expression of PCNA, CDK4 and BCL-2 as well as ↑ the expression of the proapoptotic BAX (dose-dependent manner) | |
| Zhang et al.<br>2010<br>United States; L 5 | epigallocatechin gallate **(EGCG)** in various concentrations (0, 1.0, 50, 100 and 200µm) or 1.25mg EGCG/day | in-vitro study- the Eker rat tumor-derived uterine leiomyoma cells treated with various concentrations of EGCG<br>Animal model- Female athymic nude Harlan Sprague Dawley mice: control group (*n* = 10) and experimental group (*n* = 10) fed with 1.25mg EGCG /mouse/day | * EGCG significantly inhibited cell proliferation in UFs (at ≥50µM EGCG significantly ↓PCNA and Cdk4 protein levels)<br>* EGCG treatment with ≥ 50 µM significantly ↑ cell apoptosis in UFs in vitro and in vivo (determined by TUNEL technique)<br>* EGCG treatment dramatically ↓ volume and weight of tumors at 4 and 8 weeks post-treatment. | |
| Tuzcu et al.<br>2010<br>Japan; L 5 | diet containing 0.048 mg **selenium (Se)**/kg or the diet supplemented with 0.2 mg or 0.4 mg of **Se**/kg of diet. | Animal model- Japanese quail<br>No 180 | * Dietary supplementation with selenium ↓ the size of spontaneously occurring leiomyoma of the oviduct with no effects on number of tumors | |
| Tsuiji et al.<br>2011<br>Japan; L 5 | **Curcumin** | Eker rat-derived uterine leiomyoma cell lines (ELT-3 cells) | *Curcumin: inhibitory effects on UF cell proliferation through activation of peroxisome proliferator-activated receptor-gamma (PPARγ) | |
| Roshdy et al.<br>2013 (2010-2011)<br>Egypt; L 1 | green tea extract (epigallocatechin gallate [**EGCG**]) – 800 mg/day (2 capsules) vs placebo | 39 reproductive-age women (age 18–50 years)<br>placebo group (n = 11), | * Green tea extract intake significantly ↓UF volume<br>* EGCG significantly ↓ fibroid-specific symptoms | *UF volume assessed by ultrasonography |

| | | | | |
|---|---|---|---|---|
| | | study group (n = 22) | * EGCG significantly improved health-related quality of life (HRQL)<br>* No adverse effects of treatment with EGCG | |
| Zhang et al.<br>2014<br>United States; L 5 | 0 or 100 μM of **EGCG** | in-vitro study - wild-type human leiomyoma (WT-HuLM) | *Green tea extract inhibited proliferation of human leiomyoma cells (mediated via catechol-O-methyltransferase) | |
| Ahmed et al.<br>2016<br>United States; L 5 | **prodrug of EGCG** and prodrugs of EGCG analogs (**pro-EGCG analogs**) | in-vitro study-the human leiomyoma cell line (HuLM) | *Pro-EGCG analogs in comparison with Pro-EGCG have enhanced antiproliferative, anti-angiogenic (inhibited expression of vascular endothelial growth factor receptor 2- VEGF-R and vascular endothelial growth factor C- VEGF-C), and antifibrotic properties in HuLM | *Prodrug of EGCG (Pro-EGCG) and prodrugs of EGCG analogs (pro-EGCG analogs) show increased stability |
| Yu et al.<br>2019<br>China; L 5 | Combination of herbs (***Rhizoma Curcumae* and *Rhizoma Sparganii)***<br>(6.67 g/kg) | Animal model- Sprague Dawley rats No 30 randomly divided into 3 groups: control, UL and UL and CRSR therapy | * UFs in rats significantly shrunk after CRSR therapy<br>* CRSR regulated crucial signaling pathways in UFs formation (as PPAR, MAPK, TGF-beta/Smad, and Notch signaling pathways)<br>* CRSR regulated the signaling pathways related to metabolism of cofactors and vitamins, nucleotide and amino acids metabolism<br>* CSCR directly suppressed ECM metabolism<br>* Serum P and $E_2$ levels significantly ↓ after CRSR | |
| Porcaro et al.<br>2020<br>Italy; L 1 | Treatment with two tablets of 150 mg **EGCG** + 25 µg vitamin D + 5 mg vitamin B6 daily, for 4 months | 30 symptomatic women with myomas (aged 28- 46 years) divided in two groups:<br>Study group (n=15)- treated with EGCG and Vit D tablets<br>Control group (n=15)- received no treatment. | * Total myoma volume significantly ↓ after combination of EGCG, vit D and vit B6 treatment<br>* Total myoma volume significantly ↑ in the control group<br>* Improvement in quality of life (QoL) along with reduction of myoma-related symptoms observed in women treated with EGCG, vit D and vit B6 | |
| Greco et al.<br>2020<br>Italy; L 5 | **quercetin** and indole-3-carbinol (**I3C**) | in-vitro study-leiomyoma cells treated with quercetin or indole-3-carbinol at different concentrations (10 µg/ml; 50 µg/ml; 100 µg/ml; and 250 µg/ml) for 48 h | * Quercetin and I3C have anti-fibrotic properties due to the influence on mRNA and protein expressions of ECM (↓collagen 1A1 and fibronectin)<br>* Quercetin and I3C treatment significantly ↓ migration of leiomyoma cells<br>* Leiomyoma cell proliferation not affected by quercetin and I3C | |

| | | | | |
|---|---|---|---|---|
| Grandi et al.<br>2021<br>Italy; L 1 | tablets containing **EGCG** 150 mg, Vitamin B6 5 mg and Vit D 25 µg - 2 tablets per day for 3 months (total dose: EGCG 300 mg, Vitamin B6 10 mg and VD 50mg/day) | 16 premenopausal women (age 43-52 years) | * Treatment with EGCG and Vitamin B6 and Vitamin D induced significant ↓ in UF size and menstrual flow length in women in late reproductive life<br>* No significant changes observed in health-related QoL (SF-36 questionnaire) and quality of sexual life (FSFI questionnaire) | *UFs diagnosed by TVS (diameter 3-10 cm). |
| Biro et al.<br>2021<br>Germany; L 1 | 3 capsules daily of green tea extract (GTE) containing: **EGCG** 390 mg, Vitamin C 60 mg, Piperine 15 mg and Caffeine 3 mg | 25 Caucasian race participants (age 33-49 years) | *No significant changes in myoma size after GTE<br>* Significant improvement in physical cumulative score; no effect on mental QoL score after GTE<br>* No significant improvement in global QoL score<br>* No significant changes in symptoms after GTE<br>* No relevant adverse side effects after GTE | *Quality of life (QoL) assessed by SF-12 questionnaire<br>*Size of the myomas evaluated by TVS |
| Miriello et al.<br>2021<br>Italy; L 3 | Treatment with tablets containing 150 mg **EGCG**+ 25 µg vitamin D + 5 mg vitamin B6 (two tablets daily for 4 months) | 95 women of reproductive age with ≥ 1 myoma<br>Two groups:<br>Study (No 41)- treated with EGCG + vit D + vit B6 for 4 months;<br>Control (No 54) received no treatment. | *Combined supplementation of EGCG, vit D and vit B6 significantly ↓myoma volume (by 37.9%)<br>* Significant ↓ of peripheral myoma vascularization after treatment<br>* Statistically significant improvement of myoma-related symptoms after treatment<br>* Significant improvement in patients' quality of life and health noted after treatment<br>* No side effects noted after treatment | *Volume and vascularization of myomas analyzed by USG<br>*Quality of life and health evaluated by questionnaires: SF-36 and PGI-I |
| Feng et al.<br>2021<br>China; L 5 | The herbs (***Rhizoma Curcumae*** (RC): ***Rhizoma Sparganii*** (RS) = 1:1) | Animal model- Sprague Dawley (SD) rats No 72<br>groups: control group, RCRS-treated groups (66.7- 6,67 g/kg/day; 33.3- 3.33 g/kg/day; 16.7%- 1.67 g/kg/day), RC-treated group, RS-treated group. | * Treatment with RCRS effective in rats (prevention and treatment of UFs)<br>* RCRS significantly ↓ expression of ECM component collagen I, fibroblast activation protein, and transforming growth factor beta (TGF-β) and ↓expression levels of signal factors (AKT, ERK, and MEK) in cell proliferation-related pathway | |

**Table S1.** Influence the dietary compounds on the uterine fibroids.
